# Supplementary material for: Structural characterization of HIV-1 matrix mutants implicated in envelope incorporation
Source: J Biol Chem. 2021 Jan 22;296:100321. doi: 10.1016/j.jbc.2021.100321 (PMC7952133; doi:10.1016/j.jbc.2021.100321)
Supplement: Figures S1 to S4 [file mmc1.pdf]

Supporting Information

Structural characterization of HIV-1 matrix mutants implicated in envelope incorporation

Gunnar N. Eastep, Ruba H. Ghanam, Todd J. Green, and Jamil S. Saad[1]*

Running Title: HIV-1 matrix mutants that impact envelope incorporation

Department of Microbiology, University of Alabama at Birmingham, Birmingham, AL 35294

Corresponding Author:

Jamil S. Saad, Ph.D.
Department of Microbiology
University of Alabama at Birmingham
845 19th Street South, Birmingham, AL 35294
Phone: (205)-996-9282
Email: saad@uab.edu

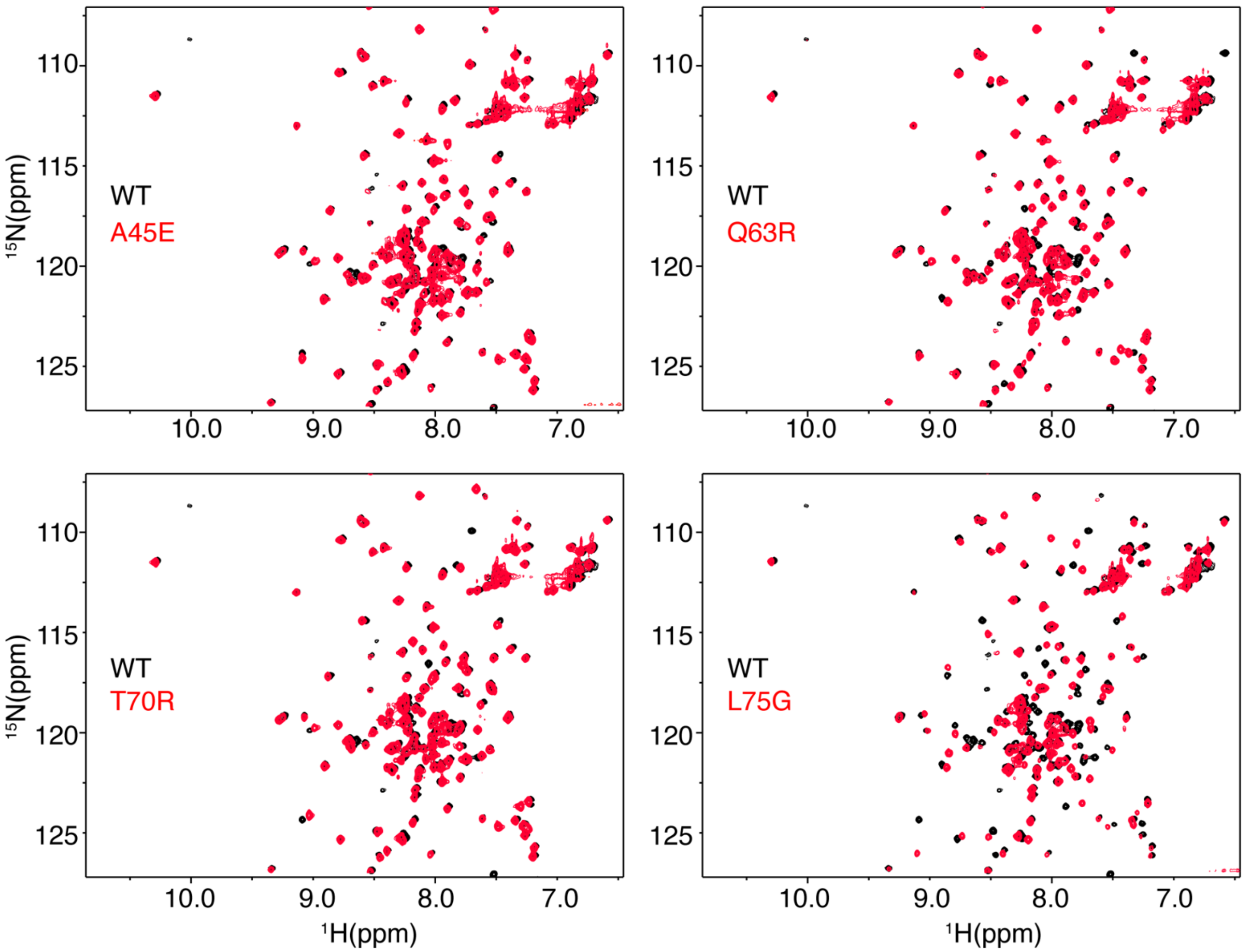

Fig. S1. Overlay of 2D ^{1}H-^{15}N HSQC spectra obtained for WT and mutant HIV-1 MA proteins at 150 μM (308 K) in a buffer containing 50 mM sodium phosphates (pH 6) and 50 mM NaCl.

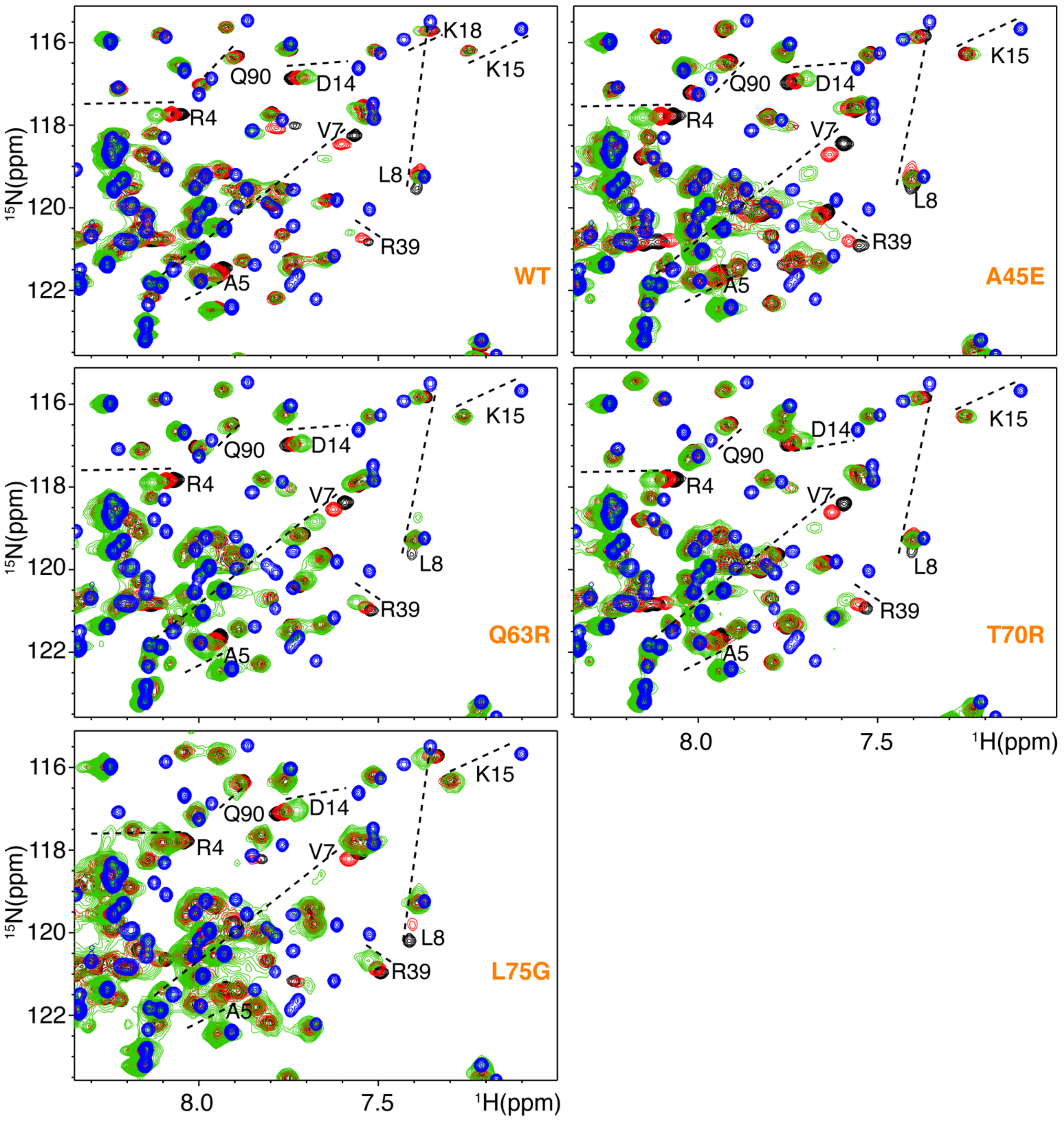

Fig. S2. Overlay of 2D ^{1}H-^{15}N HSQC spectra obtained for WT and mutant HIV-1 MA proteins at 50 µM (black), 150 µM (red), and 450 µM (green) (308 K) in a buffer containing 50 mM sodium phosphates (pH 5.5) and 50 mM NaCl. Signals that progressively shift towards those of the corresponding myr(–)MA protein (blue spectrum) are labeled. Dashed line indicates the direction of the resonance shifts towards the corresponding blue signal.

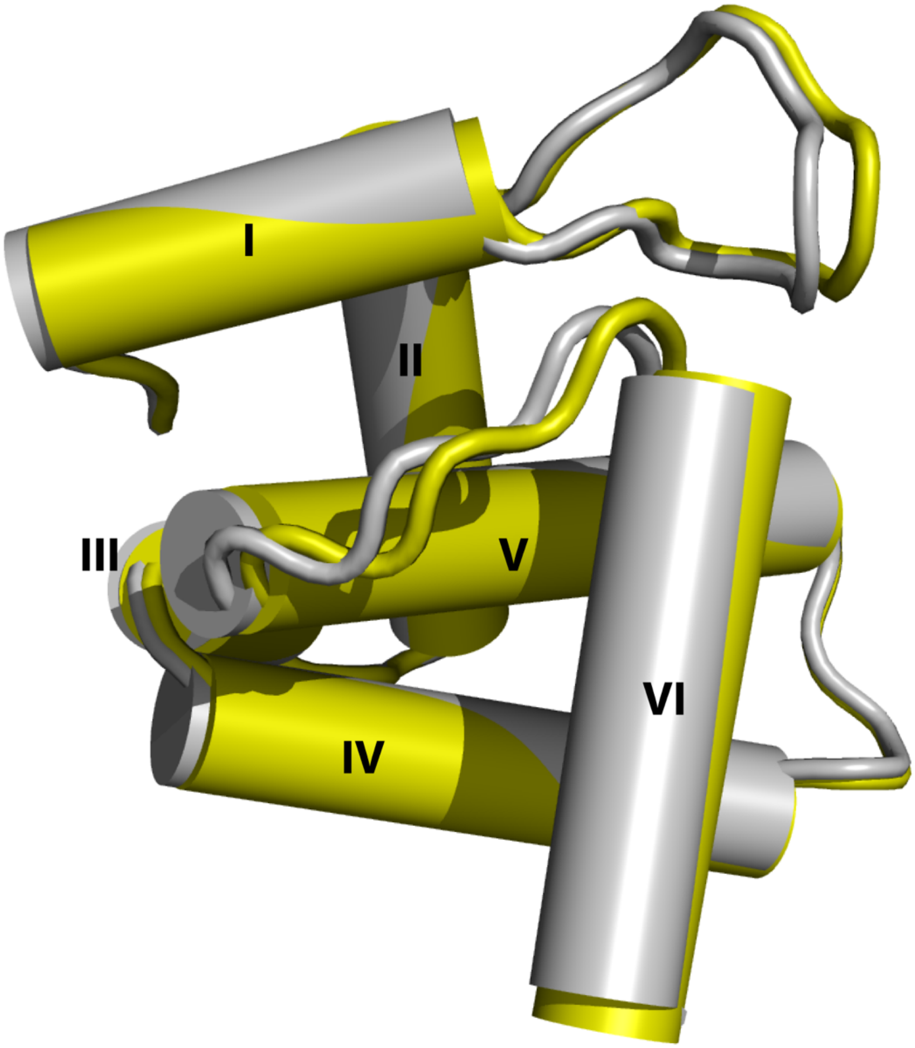

Fig. S3. Superimposition of HIV-1 myr(–)MA Q63R structures from crystals form 1 (PDB 7JXR; yellow) and form 2 (PDB 7JXS; grey). Residues 110–132 lack secondary structure and are not shown for clarity. Ribbon representations of the structures were generated using the PyMOL molecular graphics system (Schrödinger, LLC).

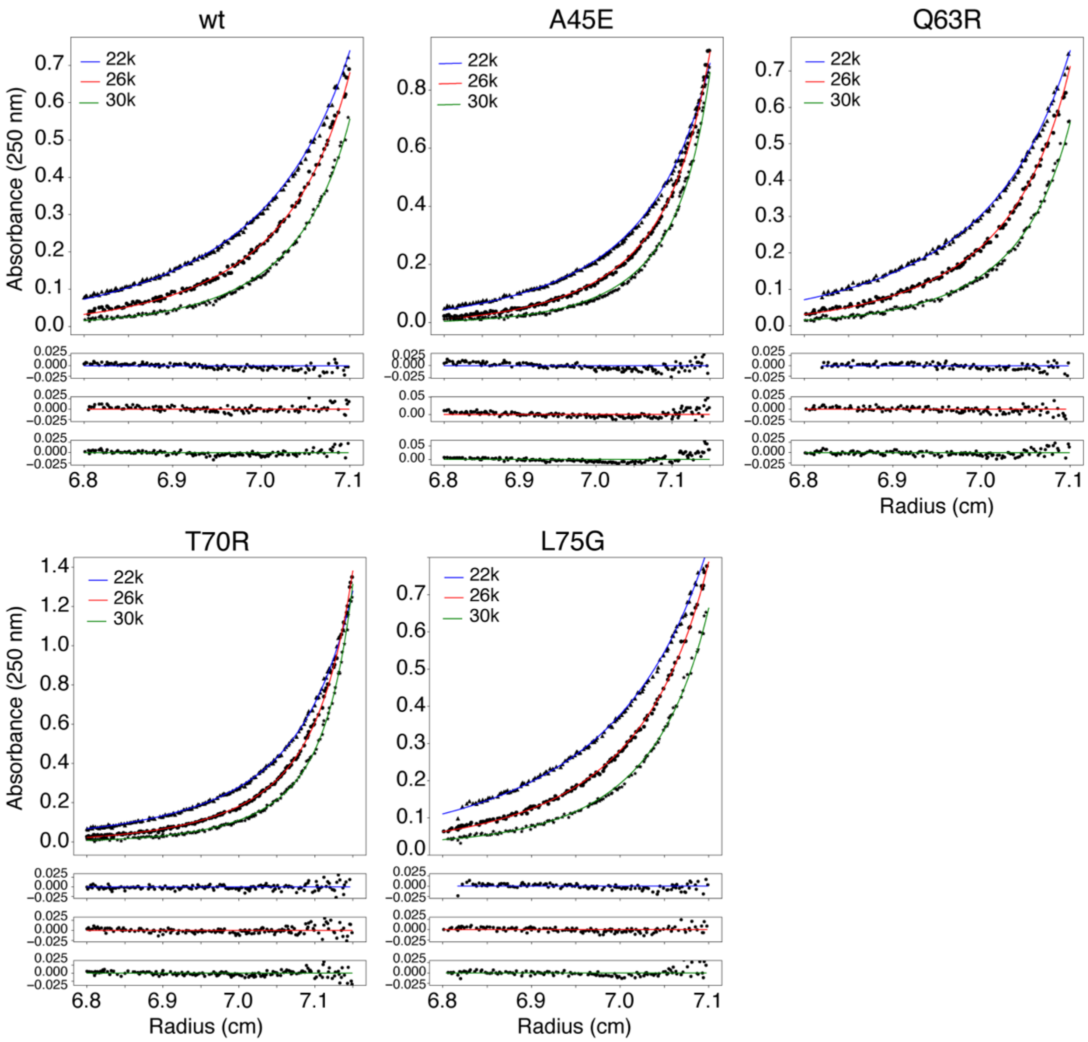

Fig. S4. Representative sedimentation equilibrium profiles obtained for WT and mutant MA at 22000, 26000, or 30000 rpm [(20 °C, ~70 μM)] at pH 5.5. Sedimentation profiles fit best to monomer–trimer equilibrium. Dots represent experimental data and solid lines represent the result of the global fit. Bottom plot show residuals resulting from the global fit.
